# The integrated nuclear medicine and radiology residency program in the Netherlands: strengths and potential areas for improvement according to nuclear medicine physicians and radiologists

**DOI:** 10.1007/s00259-022-05699-8

**Published:** 2022-02-23

**Authors:** Ton Velleman, Thomas C. Kwee, Rudi A. J. O. Dierckx, Yfke P. Ongena, Walter Noordzij

**Affiliations:** 1 Medical Imaging Center, Departments of Radiology, Nuclear Medicine and Molecular Imaging, University Medical Center Groningen, University of Groningen, Hanzeplein 1, P.O. Box 30.001, 9700 RB Groningen, The Netherlands; 2 Faculty of Arts, Communication and Information Sciences, University of Groningen, Groningen, The Netherlands

**Keywords:** Internship and residency, Nuclear medicine, Radiology, Training programs

## Abstract

**Purpose:**

To evaluate the Dutch integrated nuclear medicine and radiology residency program from the perspective of nuclear medicine physicians and radiologists.

**Methods:**

A survey was distributed among nuclear medicine physicians and radiologists in hospitals that participate in the Dutch integrated nuclear medicine and radiology training program.

**Results:**

A total of 139 completed questionnaires were included. Nuclear medicine physicians (*n* = 36) assigned a mean score of 5.7 ± 2.0, and radiologists (*n* = 103) assigned a mean score of 6.5 ± 2.8 (on a 1–10 scale) to the success of the integrated training program in their hospital. On multiple regression, female gender of the survey participant (*B* = 2.22, *P* = 0.034), musculoskeletal radiology as subspecialty of the survey participant (*B* = 3.36, *P* = 0.032), and the survey participant’s expectancy of resident’s ability to handle workload after completion of residency were significantly associated with perceived success of the integrated training program (*B* = 1.16, *P* = 0.023). Perceived strengths of the integrated training program included broadening of expertise, a better preparation of future imaging specialists for hybrid imaging, increased efficiency in training residents, and increased efficiency in multidisciplinary meetings. Perceived weaknesses of the integrated training program included reduced exposure to nuclear medicine, less time for research and innovation, and concerns about its international recognition.

**Conclusion:**

This study provided insights into the experiences of nuclear medicine physicians and radiologists with the Dutch integrated nuclear medicine and radiology residency program, which may be helpful to improve the program and similar residency programs in other countries.

**Supplementary Information:**

The online version contains supplementary material available at 10.1007/s00259-022-05699-8.

## Introduction

Nuclear medicine uses an ever increasing arsenal of radio-labeled compounds for various diagnostic and therapeutic indications [1]. Hybrid imaging, which is the combination of single photon or positron emission tomography (PET) with computed tomography (CT) or magnetic resonance imaging (MRI), has facilitated the correlation between metabolic processes and anatomy. One of many other important areas of nuclear medicine is the rapidly developing field of thera(g)nostics, which combines diagnostic and therapeutic applications [2]. With new, complex developments in imaging techniques and in diagnostic and therapeutic radiotracers, new challenges arise in daily practice in the communication with clinicians, in multidisciplinary consultation, and also in the way residents are trained.

Several nuclear medicine residency training programs have integrated (parts of) nuclear medicine with radiology training programs [3]. This was also the case for the Netherlands, where the separate residency programs for nuclear medicine and radiology have been replaced by a completely integrated residency training in 2015 [4]. This Dutch integrated training program consists of 2.5 years of general nuclear medicine and radiology, followed by another 2.5 years of training of which 1.5 years in a subspecialty of the resident’s choice. There are eight subspecialties for the residents to choose from, of which seven radiology-based and one nuclear medicine. The latter subspecialty is named “nuclear medicine and molecular radiology” (NMMR). A resident that successfully completes the integrated training and the NMMR subspecialty will bear the title “nuclear radiologist.” The societies of radiology and nuclear medicine in the Netherlands implemented the integrated training program in 2015, with the aim to ensure quality and efficiency of reporting medical imaging and communication with colleagues from other medical specialties [4].

More than 5 years have elapsed since its implementation, which provides the opportunity to evaluate firsthand, and independently from the organizing professional societies, the experiences from nuclear medicine physicians and radiologists who work in hospitals that offer this integrated training program. Previous research has identified factors that influence a resident’s choice to start the NMMR subspecialty of the integrated training, but experiences from nuclear medicine physicians and radiologists about the integrated training are still lacking [5]. This information may be useful to identify strengths of the program that may be emulated by other national societies who consider developing a similar program, and on the other hand, identify potential areas for improvement. Hence, the purpose of this study was to evaluate the Dutch integrated nuclear medicine and radiology residency program from the perspective of nuclear medicine physicians and radiologists.

## Materials and methods

### Medical ethics review board

The local medical ethics review board approved this prospective survey study (IRB number: 202000862).

### Participants

All nuclear medicine physicians and radiologists who work in hospitals that participate in the integrated nuclear medicine and radiology training program in the Netherlands were eligible to participate in this survey. Note that there are eight academic training hospitals and 28 non-academic training hospitals, in eight geographically designated residency training regions in the Netherlands. The curriculum of the integrated training program has been established by the nuclear medicine and radiology societies of the Netherlands and is therefore the same in all of these hospitals; the Dutch integrated training curriculum is summarized in Supplemental Table 1 [4].

Eligible nuclear medicine physicians and radiologists were contacted by e-mail. This e-mail contained a brief explanation of the study purpose and a request to participate. Participation in this survey was voluntary. Access to the survey was made possible via a digital link which was provided in the email.

The initial request to participate was sent by email on the 23rd of December 2020, and a reminder was sent on the 6th of January 2021. Inclusion took place between the 23rd of December and the 15th of February.

Partially completed questionnaires were excluded from the analysis as well as the group of participants who practice both nuclear medicine and radiology (dual practitioners) due to the small size of this group.

### Survey

The questionnaire was developed and analyzed by radiologists (T.V. and T.C.K.), a nuclear medicine physician (W.N.) and a survey specialist (Y.O.), and was in part based on previous literature [6]. The survey was digitalized using the Qualtrics Core XM survey software (Qualtrics, LLC an SAP America Inc. Company).

The questionnaire contained 20 closed-ended questions and 6 open-ended questions. The closed-ended questions captured basic characteristics of the participants, including age, gender, type of training curriculum followed, specialty (nuclear medicine or radiology) and subspecialty, the type of center were they followed their own training, (academic, non-academic or a combination of both), the national region they were trained in and are currently practicing in, the type of hospital where they currently practice (academic, non-academic or both), and years of post-residency experience (as nuclear medicine physician or radiologist); the closed-ended questions are summarized in Supplemental Table 2. The closed-ended questions also addressed the participants’ perceived rate of integration of the nuclear medicine and radiology departments in their hospital (on a 10-point scale), and if there have been residents in training for the NMMR subspecialty in their hospital, the possibility for residents to write combined clinical reports for hybrid imaging (e.g., PET-CT) and the supervisor of these combined reports (nuclear medicine physician, radiologist or both) in their hospital, time allocated to residents to perform research in their hospital (yes/no), the perceived distribution of allocated time between nuclear medicine and radiology in the first 2.5 years of training (balanced, unbalanced and in favor of nuclear medicine, or unbalanced and in favor of radiology) in their hospital, estimated chance of future employment of residents after completion of training (5-point scale from 1 slim to 5 excellent), expected chance of recognition of the training program by other European countries (5-point scale from 1 unlikely to 5 very likely), expectancy of resident’s ability to handle workload in clinical practice after completion of residency (5-point scale from 1 unlikely to 5 very likely), and level of independence of senior residents (5-point scale from 1 minimal to 5 complete).

The open-ended questions evaluated the perceived success of the integrated training program in their hospital (on a scale from 0 to 10), their general opinion on the integrated training program, and the opportunity to give additional comments on the closed-ended questions. The data collected from the open-ended questions were analyzed to find common categories of strengths and weaknesses of the integrated training program perceived by nuclear medicine physicians and radiologists. The answers on the closed-ended and open-ended questions are summarized in Table 1 and Table 2.

**Table 1:** Characteristics of nuclear medicine physicians (NM) and radiologists (RAD) who participated in the survey

Variable	NM (*n* = 36)	RAD (*n* = 103)
Age (years)		
31–40/41–50/51–60/60 + /NI	9/13/8/6/0	33/35/25/9/1
Gender		
Male/female/NI*	21/15/0	72/30/1
Training curriculum followed by participants		
Integrated/old style nuclear medicine^*^/old style radiology^*^/older/NI*	0/34/1/0/1	6/1/58/38/0
Type of center where survey participant’s residency training was done		
Academic/non-academic/combination/other	24/7/5/0	34/22/47/0
Postresidency experience (years)		
0–10/11–20/21–30/30 + /NI*	15/14/5/2/0	51/26/20/3/3
Current hospital of practice		
Academic/non-academic/combination/other	15/19/1/1	41/58/4/0
Resident’s currently or previous in training with NMMR subspecialty		
Yes/no/do not know/NI*	20/7/2/7	58/21/4/20
Perceived rate of integration of nuclear medicine and radiology departments		
None/low/mid/high/fully/NI*	2/4/9/11/9/1	1/13/24/31/30/4
Perceived rate of success of the integrated training***		
Failure/low/mid/high/success/NI*	0/5/12/9/1/9	0/14/17/34/13/25
Multidisciplinary meeting attendance by		
Only radiologist/only nuclear medicine physician/both/otherwise/NI*	0/0/18/17/1	8/1/54/39/1
Sufficient time for residents to do research		
Yes/no/NI*	16/18/2	91/12/0

^*^NI, not indicated; *NMMR*, nuclear medicine and molecular radiology

^**^Old style refers to previous nuclear medicine and radiology training programs which were largely separated

^***^The 10 punt scale that was used is summarized in steps of 2 grades, ranging from failure as the lowest, up until success for the highest, for easier overview

**Table 2:** Association between different variables (characteristics of nuclear medicine physicians and radiologists, their hospitals, and their opinion on different aspects the integrated training) with the perceived success of the integrated training on multiple regression

Variable	Beta-coefficient	95% *CI*	*P*-value
Age (years)	− 0.038	[− 0.39–0.32]	.794
Gender (male or female)	2.22	[0.25–4.19]	.034^3^
Received type of training (integrated vs. previous)	0.25	[− 1.23–1.73]	.683
Hospital of training (academic vs. non-academic)	− 2.46	[− 5.25–0.34]	.074
Region of training^1^	0.14	[− 0.38–0.65]	.523
Years of post-residency experience	− 0.03	[− 0.41–0.35]	.841
Specialty (nuclear medicine or radiology)	2.02	[− 1.11–5.15]	.158
Subspecialty^2^	3.36	[0.43–6.30]	.032^4^
Region of practice^1^	− 2.6	[− 7.12–1.93]	.201
Hospital of practice (academic vs. non-academic)	1.75	[− 2.25–5.75]	.313
Rate of integration of departments (scale 0–10)	0.42	[− 0.33–1.16]	.208
NMMR residents in training in hospital (yes/no)	− 1.83	[− 4.98–1.33]	.198
Combined reporting by resident (yes/no)	0.52	[− 3.68–4.72]	.763
Combined reporting supervision (nuclear medicine physician, radiologist or both)	− 0.42	[− 2.69–1.85]	.653
Multidisciplinary meeting attendance (nuclear medicine physician, radiologist, or both)	0.55	[− 2.15–3.25]	.624
Sufficient time for research (yes/no)	1.36	[− 1.79–4.51]	.318
Distribution of allocated time between nuclear medicine and radiology in the first 2.5 years of training (balanced vs. unbalanced)	− 0.41	[− 2.51–1.69]	.636
Future employment chances for residents (scale 0–5)	0.63	[− 0.80–2.05]	.310
Recognition of the training in the European Union (scale 0–5)	0.10	[− 1.06–1.26]	.831
Ability of residents to handle workload after completion of residency (scale 0–5)	1.16	[0.24–2.09]	.023
Independence of senior residents (scale 0–5)	− 0.71	[− 2.55–1.14]	.370

^1^Eight different geographical training and practice regions in the Netherlands

^2^Nuclear medicine and molecular radiology and seven radiology subspecialties

^3^Females were significantly more positive regarding the perceived success of the integrated training program

^4^Participants in the musculoskeletal subspecialty were significantly more positive regarding the perceived success of the integrated training program compared to those in the abdominal subspecialty (reference category)

### Data analysis

The data collected from the closed-ended questions were compared and analyzed via a multiple regression analysis. We used the participants’ answer to the question “what is your opinion about the success of the integrated training?” (ranging from failure to a complete success on a scale from 0–10) as the dependent variable. The remaining closed-ended questions were entered as independent variables. Multiple regression analysis was used to explore a link between all predictors and their additive effects on the dependent variable. *P*-values less than 0.05 were considered statistically significant. The data collected from the open-ended questions were descriptively analyzed. Statistical analyses were performed using R Studio, Version 1.2.5033.

## Results

### Survey

A total of 759 potentially eligible participants were contacted by email (54 nuclear medicine physicians and 714 radiologists). A total of 184 questionnaires were digitally returned (response rate of 24%). Forty partially completed questionnaires and 5 questionnaires of participants with a dual certification in nuclear medicine and radiology were excluded. A total of 139 fully completed surveys remained for inclusion. No nuclear medicine physician and only 6 radiologists participating in this survey had undergone the integrated training themselves.

### Participants and training center characteristics

The 139 included questionnaires were completed by 36 nuclear medicine physicians and 103 radiologists. Each of the eight residency training regions in the Netherlands was represented among the participants. The nuclear medicine physicians assigned a mean score of 5.7 ± 2.0, and the radiologists assigned a mean score of 6.5 ± 2.8 (on a 1–10 scale) to the success of the integrated training in their hospital. The basic characteristics of the participating nuclear medicine physicians and radiologists are summarized in Table 1, and median values of the continuous variables in Supplemental Table 3.

### Association between variables and perceived success of the integrated training program

On multiple regression, female gender of the survey participant (*B* = 2.22, *P* = 0.034), musculoskeletal radiology as subspecialty of the survey participant (*B* = 3.36, *P* = 0.032), and the survey participant’s expectancy of resident’s ability to handle workload after completion of residency (*B* = 1.16, *P* = 0.023) were significantly associated with perceived success of the integrated training program (Table 2).

### Strengths and weaknesses of the integrated training program according to nuclear medicine physicians

Nuclear medicine physicians described varying degrees of integration (ranging from “complete” to “just on paper”) of management, staff, resident training, and research between nuclear medicine and radiology departments.

Nuclear medicine physicians made 31 comments about the value of the integrated training program (9 strengths, 5 neutral comments, and 17 weaknesses). Advantages of the integrated training program that were mentioned were increased expertise in hybrid imaging reporting, broadening of competencies and expertise for nuclear medicine and radiology residents, and increased efficiency for multidisciplinary meetings (i.e., only one staff member required who can interpret both modalities). Weaknesses of the integrated training program that were mentioned were concerns about its international recognition, the expected mandatory fellowship after the integrated training, the lack of internal medicine training for NMMR subspecialty residents (note that 1 year of internal medicine training was included in the previous training program), whether or not the Dutch society of nuclear medicine can remain a member of the EANM, the lack of exposure to nuclear medicine, reduced time for innovation and research related to nuclear medicine due to the integration of two training programs into one, and the added radiology “workload” compared to the previous training programs. Some participants worried that the aforementioned factors might turn NMMR training less attractive for new residents.

### Strengths and weaknesses of the integrated training program according to radiologists

Radiologists described varying degrees of integration between nuclear medicine and radiology departments in their hospitals regarding finance, management, staff, resident training and research, and work activities.

Radiologists made 71 comments about the value of the integrated training program (40 strengths, 8 neutral comments, and 23 weaknesses). Strengths of the integrated training program that were mentioned were integration leads to better preparation of future medical imaging specialists with the expected increase in hybrid imaging and imaging directed on pathology/organs and therapies, increased efficiency with combined reporting and in multidisciplinary meetings, better patient care, broadening of expertise for both nuclear medicine physicians and radiologists, and an opportunity to learn from each other. Weaknesses of the integrated training program that were mentioned were less time for residents to reach the same level of quality, decreased exposure and knowledge of nuclear medicine and radiology compared to the previous training programs, not all competencies can be acquired to an independent level for residents due to the sheer number of available competencies, integration of departments goes slow which leads to less success of the integrated training, less time to do research and delve into new developments of nuclear medicine and radiology (thera(g)nostics/big data/artificial intelligence), possible depreciation of Dutch nuclear medicine and radiology on a European level, and concerns that the completed training is not necessarily equal to the previous training programs.

### Suggested points for improvement

The nuclear medicine physicians and radiologists made several suggestions for improvement of the integrated training program. These included more time for exposure to nuclear medicine (both nuclear medicine physicians and radiologists), especially during the first 2.5 years of training and for residents who choose the NMMR pathway to comply with European standards (nuclear medicine physicians), increased time for innovation and research in nuclear medicine for residents during their training (nuclear medicine physicians), improved workflow integration of both departments (both nuclear medicine physicians and radiologists), and fusion of the nuclear medicine and radiology societies (radiologists). Some suggested increasing the duration of the training from 5 to 6 years (both nuclear medicine physicians and radiologists).

## Discussion

In 2015, the Netherlands started with a unique integrated nuclear medicine and radiology residency training program. Now, 5 years later, this study for the first time looks at its strengths and weaknesses. All nuclear medicine physicians and radiologists that participate in the integrated training program in the Netherlands were contacted via email to complete this study survey. Eventually, we included 36 nuclear medicine physicians and 103 radiologists. The survey participants assigned average marks of 5.7 (nuclear medicine physicians) and 6.5 (radiologists) on a 10-point scale to the success of the integrated training program, suggesting both that the program is feasible and that there is still room for improvement. Our data also showed that nuclear medicine physicians and radiologists were significantly more positive about the integrated training program when they were female, musculoskeletal radiologists, and when they deemed residents in their hospital capable to handle workload after completion of residency. The reason why women and musculoskeletal radiologists were significantly more positive about the integrated training program than their male and other subspecialty counterparts remains unclear. Further in-depth interviews with these populations are necessary to understand their more positive attitude towards the integrated training program. On the other hand, It seems plausible that nuclear medicine physicians and radiologists were significantly more positive about the integrated training program when they considered their residents able to handle workload after completion of residency, because one of the main goals of any residency program should be to deliver specialists who are directly deployable in busy clinical practice. Clinical productivity of residents may be monitored with metrics such as relative value units. Of interest, previous research has shown that clinical productivity is independently associated with medical knowledge relevant to radiology practice during radiology residency [7]. Further research is necessary to understand how residents can be best selected and trained to reach a level of clinical productivity that prepares them well for post-residency work life. None of the other variables that were investigated were significantly independently associated with the perceived success of the integrated training program.

Of interest, around three-quarters of participants additionally expressed their opinion about the success of the integrated training in the open-ended question. This is in line with the attention this topic has received in the respective professional societies in the Netherlands. Both nuclear medicine physicians and radiologists agreed that positive aspects of the integrated training were broadening of expertise, a better preparation of future imaging specialists for the expected increase in hybrid imaging, increased efficiency in training residents, and increased efficiency in multidisciplinary meetings. Weaknesses of the integrated training program mentioned by participants in this survey included reduced exposure to nuclear medicine, less time for research and innovation compared to the previous training programs, and concerns about its international recognition.

Several nuclear medicine physicians and radiologists who participated in this survey provided suggestions to improve the training, which included allocating more time during the first 2.5 years of training to nuclear medicine and for residents who chose the NMMR pathway to comply with European standards, allocating more time for innovation and research, and increasing integration of departments and workflows.

A previous survey study, in which 114 residents participated, investigated reasons that influence a resident’s choice for the nuclear medicine subspecialty in the Dutch integrated training program [5]. The results of that previous study and those of the current study show that both residents, nuclear medicine physicians and radiologists, consider increased expertise, efficiency of training, broadening of competencies, and a better preparation for the expected increase in hybrid imaging in the future, as strengths of the integrated training program. Furthermore, both residents, nuclear medicine physicians and radiologists, generally indicated poor integration of nuclear medicine and radiology departments, imbalance in exposure to the detriment of nuclear medicine, and concerns about its international recognition, as weaknesses of the integrated training program. However, unlike residents, nuclear medicine physicians and radiologists generally expressed concerns about reduced time for research and loss of quality of the trained residents. Meanwhile, unlike nuclear medicine physicians and radiologists, residents were generally concerned about their future employment prospects. This difference in concerns might be due to differences in interests between future employment and quality of trained residents.

Above-mentioned factors may be taken into account by policy makers and other stakeholders who are developing and aiming to improve integrated nuclear medicine and radiology residency programs.

This study had some limitations. First, the ratio of nuclear medicine physicians to radiologists who participated in this survey (36:103) was relatively high compared to the ratio of nuclear medicine physicians to radiologists comprising the entire workforce in all training hospitals in the Netherlands (54:714). This relatively higher participation rate by nuclear medicine physicians might potentially be explained by the possibility that the integrated training affects nuclear medicine more than radiology. Relatively less time is available for nuclear medicine compared to radiology during the integrated training and also compared to the previous largely separate nuclear medicine training program, which might raise increased concerns from nuclear medicine physicians and in turn have led to a better response rate. In other countries where different forms of integrated training programs are running, similar concerns among nuclear medicine physicians exist [8]. Second, the integrated training was implemented in 2015 and only a few participants have followed the integrated training as residents themselves. Not all participants may be completely used to this form of training and time may be needed to fine-tune implementation, to build experience and gain more widespread acceptance. Third, with promising developments in both fields, such as artificial intelligence and thera(g)nostics, a change in clinical practice may be expected which might also lead to a change in how the integrated training is evaluated. Further research is needed to investigate the impact of the integrated training on the vast number of diagnostic and therapeutic nuclear medicine procedures in daily practice. Fourth, the response rate was slightly on the lower normal end of what can be expected from survey results in healthcare [9]. Fifth, the response rate of the radiologists was relatively low compared to the nuclear medicine physicians. It remains unclear if this was caused by differences in interest to participate in this survey or due to other differences inherent to both specialties. And last, the training curriculum defines that only nuclear radiologists will be trained in radionuclide therapies, and will be fully certified for therapeutic applications after finishing their residency. Nuclear diagnostic procedures are carried out by any (nuclear) radiologist with the required level of entrustable professional activity. The radionuclide therapies were not specifically addressed in this survey, since there are yet only small numbers of fully trained nuclear radiologists. Further research might provide more insights on how this will impact the distribution of nuclear medicine activities.

In conclusion, this study provided insights into the experiences of nuclear medicine physicians and radiologists with the Dutch integrated nuclear medicine and radiology residency program, which may be helpful to improve the program and similar residency programs in other countries.

## Supplementary Information

Below is the link to the electronic supplementary material.

Supplementary file1 (DOCX 15 KB)

Supplementary file2 (DOCX 14 KB)

Supplementary file3 (DOCX 15 KB)

## Data Availability

All data was processed anonymously and only accessible to the main author.

## Code availability

Qualtrics Core XM survey software (Qualtrics, LLC an SAP America Inc. Company).

## Declarations

### Clinical trial registration

Not applicable. A waiver was provided by the local medic ethics review board.
