# Supplementary material for: The integrated nuclear medicine and radiology residency program in the Netherlands: strengths and potential areas for improvement according to nuclear medicine physicians and radiologists
Source: Eur J Nucl Med Mol Imaging. 2022 Feb 23;49(9):3016–22. doi: 10.1007/s00259-022-05699-8 (PMC9250465; doi:10.1007/s00259-022-05699-8)
Supplement: Supplementary file 1 — Supplementary file1 (DOCX 15 KB) [file 259_2022_5699_MOESM1_ESM.docx]

**Supplemental table 1.** The Dutch integrated nuclear medicine and radiology training curriculum as established in 2015. Time allocated for each subspecialty in different phases of the training in months.

|  | Common trunk phase | Subspecialty phase^1,2^ |
| --- | --- | --- |
| Cardiothoracic | 5 | 18 |
| Abdominal | 5 | 18 |
| Intervention | 2 | 18 |
| Nuclear medicine and molecular radiology | 2 | 18 |
| Neuro- and head & neck | 4 | 12 |
| Musculoskeletal | 4 | 12 |
| Breast | 2 | 6 |
| Pediatric | 2 | 6 |
| Time for education, congress, catch-up, holiday, elective training, courses, etc. | 4 |  |
|  | 2,5 year | 2.5 year^3^ |

^1^ The total length of the training is 5 years.
^2^ Residents can either choose a subspecialty that requires 18 months or two different subspecialties
 that require 12 months and 6 months, respectively.
^3^ During the subspecialty phase a resident spends 18 months on the chosen subspecialty or subspecialties
 and 12 months on further general radiology training.
