# Supplementary material for: The integrated nuclear medicine and radiology residency program in the Netherlands: strengths and potential areas for improvement according to nuclear medicine physicians and radiologists
Source: Eur J Nucl Med Mol Imaging. 2022 Feb 23;49(9):3016–22. doi: 10.1007/s00259-022-05699-8 (PMC9250465; doi:10.1007/s00259-022-05699-8)
Supplement: Supplementary file 2 — Supplementary file2 (DOCX 14 KB) [file 259_2022_5699_MOESM2_ESM.docx]

**Supplemental table 2.** Closed- ended question from the questionnaire.

| Date of birth |
| --- |
| Gender (male or female) |
| Received type of training (integrated vs. previous) |
| Hospital of training (academic, non-academic or both) |
| Region of training |
| Years of post-residency experience as nuclear medicine physician or radiologist |
| Specialty (nuclear medicine, radiology or both) |
| Radiology subspecialty (abdominal, cardiothoracic, intervention, neuro- and head & neck, musculoskeletal, breast, pediatric, other) |
| Region of practice |
| Hospital of practice (academic, non-academic or both) |
| Possibility for residents to do NMMR subspecialty in their hospital (yes/no/other) |
| Rate of integration of departments (scale 0-10)^*^ |
| Opinion on the success of the integrated training (scale 0-10)^**^ |
| Residents currently or previous in training with NMMR subspecialty |
| Possibility for common trunk residents to do the complete NMMR subspecialty in their hospital |
| Possibility for NMMR subspecialty residents to do the complete NMMR subspecialty in their hospital |
| Combined reporting by resident (yes/no) |
| Combined reporting supervision (nuclear medicine physician, radiologist or both) |
| Multidisciplinary meeting attendance (nuclear medicine physician, radiologist or both) |
| Sufficient time for research (yes/no) |
| Distribution of allocated time between nuclear medicine and radiology in the first 2.5 years of training (balanced vs. unbalanced favoring either nuclear medicine or radiology) |
| Future employment chances for residents (scale 0-5)^***^ |
| Recognition of the training in the European Union (scale 0-5)^***^ |
| Ability of residents to handle workload after completion of residency (scale 0-5)^***^ |
| Independence of senior residents (scale 0-5)^***^ |

^*^ Ranging from 0 as no integration at all, until 10 completely integrated.

^**^ Ranging from 0 as complete failure to 10 as great success. ^***^ Ranging from 0 as very low, until 5 great.
