# Supplementary material for: The integrated nuclear medicine and radiology residency program in the Netherlands: strengths and potential areas for improvement according to nuclear medicine physicians and radiologists
Source: Eur J Nucl Med Mol Imaging. 2022 Feb 23;49(9):3016–22. doi: 10.1007/s00259-022-05699-8 (PMC9250465; doi:10.1007/s00259-022-05699-8)
Supplement: Supplementary file 3 — Supplementary file3 (DOCX 15 KB) [file 259_2022_5699_MOESM3_ESM.docx]

**Supplemental table 3.** Continuous variables with median values.

| Variable | Median | |
| --- | --- | --- |
|  | Nuclear medicine physicians | Radiologists |
| Age (years) | 48 | 45 |
| Years of post-residency experience | 13 | 10 |
| Rate of integration of departments (scale 0-10)^*^ | 7 | 8 |
| Future employment chances for residents (scale 0-5)^**^ | 4 | 4 |
| Recognition of the training in the European Union (scale 0-5)^**^ | 1 | 3 |
| Ability of residents to handle workload after completion of residency (scale 0-5)^**^ | 4 | 4 |
| Independence of senior residents (scale 0-5)^**^ | 4 | 4 |
| Success of the integrated training (scale 0-10)* | 6 | 7 |

^*^ Ranging from 0 as no integration at all, until 10 completely integrated.  ^**^ Ranging from 0 as very low, until 5 great.
